# Expression of Ice-Binding Proteins in *Caenorhabditis elegans* Improves the Survival Rate upon Cold Shock and during Freezing

**DOI:** 10.1038/s41598-019-42650-8

**Published:** 2019-05-15

**Authors:** Masahiro Kuramochi, Chiaki Takanashi, Akari Yamauchi, Motomichi Doi, Kazuhiro Mio, Sakae Tsuda, Yuji C. Sasaki

**Affiliations:** 1 Graduate School of Frontier Sciences, The University of Tokyo, Kashiwa, 277-8561 Japan; 2 AIST-UTokyo Advanced Operando-Measurement Technology Open Innovation Laboratory (OPERANDO-OIL), National Institute of Advanced Industrial Science and Technology (AIST), Kashiwa, 277-8565 Japan; 3 Molecular Neurobiology Research Group and DAI-LAB, Biomedical Research Institute, National Institute of Advance Industrial Science and Technology (AIST), Tsukuba, 305-8566 Japan; 4 Graduate School of Life Science, Hokkaido University, Sapporo, 060-0810 Japan; 5 Bioproduction Research Institute, National Institute of Advanced Industrial Science and Technology (AIST), Sapporo, 062-8517 Japan

**Keywords:** Cell biology, Molecular biology, Structural biology

## Abstract

Ice-binding proteins (IBPs) are capable of binding ice crystals and inhibiting their growth at freezing temperatures. IBPs are also thought to stabilize the cell membrane at non-freezing temperatures near 0 °C. These two effects have been assumed to reduce cold- and freezing-induced damage to cells and tissues. However, knowledge regarding the effects of IBP on the living animals is limited. Here, we characterized the relationship between the IBP effects and the physiological role by using the nematode *Caenorhabditis elegans*. The expression of fish (NfeIBPs)- and fungus-derived IBPs (AnpIBPs and TisIBP8) in *C. elegans* improved its survival rate during exposure to 0 and −2 °C (cold shock) and −5 °C (freezing). The observed cold tolerance of *C. elegans* after cold shock is attributable to the stabilization of cell-membrane lipids with IBPs, and the freezing tolerance at −5 °C can be attributed to the inhibition of ice-crystal growth by the IBPs. Significantly, the survival rate of *C. elegans* at −5 °C was improved by expression of wild-type AnpIBP and maximized by that of TisIBP8, whereas it was lowered when a defective AnpIBP mutant was expressed. These results suggest that the ice-binding ability of IBP has a good correlation with the survival rate of *C. elegans* during freezing.

## Introduction

Ice-binding proteins (IBPs) inhibit the growth of ice crystals with their surface-bound waters that are spatially organized to bind an ice crystal. Fish type I IBPs bind to a specific pyramidal plane^1^, and some type III IBPs bind to both prism and pyramidal planes^2^. These IBPs ultimately modify the crystal into a needle-like shape. In contrast, “hyperactive” IBPs identified in insects and fungi bind the prism, pyramidal, and basal planes of ice crystals, modifying the crystals into a rounded-hexagonal morphology^3^. Ice-growth inhibition is thought to reduce damage to tissues and cells of living organisms under freezing conditions. Ice-growth inhibition is closely related to the freezing point depression activity of IBP solution, which is easily evaluated as the difference between melting and freezing temperatures or “thermal hysteresis (TH)”^4,5^. In addition to these IBP-ice interactions, IBPs are also thought to stabilize cell-membrane lipids at hypothermic temperatures^6^. This membrane stabilization effect is useful for protecting cells and tissues from cold-induced damage. Actually, several fish IBPs dissolved in Euro-Collins solution significantly improved the survival rate of rat insulinoma RIN-5F cells following a 5-day incubation at 4 °C^7^. Hirano *et al*. reported that fish NfeIBPs protect cultivated mammalian cells (HepG2) during a 4 °C cold shock, suggesting that the membrane stabilization effect is correlated with TH activity^8^. Ice-growth inhibition and membrane stabilization are thought to facilitate the survival of living organisms in cold and freezing environments, and these effects of several IBPs are correlated with TH activity^8^. However, knowledge regarding the relationship between TH activity and its physiological roles in living organisms during cold shock and freezing is limited.

The nematode *Caenorhabditis elegans* is a valuable model used to characterize the functions of proteins at the behavioural and cellular level. A combination of molecular genetic manipulations and fluorescence imaging techniques has been used to identify the molecular mechanisms regulating behavioural responses and cellular functions in worms^9,10^. No ice-growth inhibiting substance, such as IBP, has been identified in *C. elegans*, but they employ several intrinsic mechanisms for survival upon lethal cold exposure. Light and pheromone-sensing neurons in *C. elegans* detect the temperature decrease in the cultivated environment and release insulin, which is received by the intestine and other neurons. The expression of DAF-16/FOXO regulating genes, which are involved in lipid production, is regulated by insulin signalling, resulting in the formation of cold tolerance in *C. elegans*^11,12^. Actually, the survival rate of worms at 2 °C cold shock was 10% or less when cultivated at 22 to 27 °C. However, most worms survived the 2 °C cold shock when cultivated at 15 °C for only 3 hours. Among nematode species, the Antarctic nematode *Panagrolaimus davidi* and *Plectus murrayi* intrinsically have strong tolerances to sub-zero and freezing temperatures^13–15^. *P. davidi* has an ice-active protein, and showed that its extracts produce an hexagonal ice crystals^16^. An expressed sequence tag (EST) analysis identified that *P. murrayi* has a protein with homology to a fish type II IBP^17^ and showed that its extracts inhibit ice-crystal growth^15^. The strategies used for survival in cold environments probably vary among nematode species. The Antarctic nematode employs a protein, such as IBP, for ice-growth inhibition, whereas *C. elegans* regulates the expression of genes involved in saturated phospholipids to protect cell-membrane lipids. Although IBPs have the potential to protect tissues and cells from cold- and freezing-induced damage, whether their effects improve the survival ability of *C. elegans* during cold shock and freezing is unclear.

Many types of IBPs have been isolated from several polar fishes, microbials, plants and insects and analysed to characterize their biochemical properties. Fish IBPs are categorized into four types (I–IV) or antifreeze glycoprotein (AFGPs). Fish type I IBPs are alanine-rich proteins consisting of a single amphipathic alpha helix and are approximately 3.3–4.5 kD in size. Type II IBPs are cysteine-rich globular proteins containing five disulphide bonds^18^. Type III IBPs exhibit overall hydrophobicity at ice binding surfaces similar to type I IBPs and are approximately 6 kD in size^19^. Type IV IBPs are alpha helical proteins rich in glutamate and glutamine, consist of a 4-helix bundle and are approximately 12 kD in size^20^. NfeIBPs isolated from *Zoarces elongatus Kner* (Notched-fin eelpout) is a type III IBP that has at least 13 isoforms. The ice-binding effect differs among its isoforms^21^. The TH activity of NfeIBP6 is approximately 0 °C (at 0.4 mM), whereas that of NfeIBP8 is approximately 0.5 °C (at 0.4 mM)^22^. Although NfeIBPs are involved in cellular protection, as previously mentioned, how these differences in TH activity affect freezing tolerance in living animals is unclear. The fungal proteins AnpIBP and TisIBP were recently isolated from *Antarctomyces psychrotrophicus*^23^ and *Typhula ishikariensis*^24^, respectively. Whether these fungal IBPs improve cold or freezing tolerance in living cells, tissues or animals remains unclear.

In this study, we investigated the functions of IBPs *in vivo* through behavioural and cellular observations of transgenic *C. elegans* expressing IBPs. We show that IBPs recently discovered in fish NfeIBPs and fungal AnpIBP improve the survival rate of worms and protect their cells from lethal cold and freezing exposure. The fungal AnpIBP T156Y mutant exhibiting low TH decreased freezing tolerance in worms at −5 °C. The fungal TisIBP8 exhibiting high TH activity was observed to dramatically improve the survival rate of worms and strongly protect muscle cells. The relationship between the ice-binding abilities of IBPs and their *in vivo* roles in tolerances and cellular protection in living animals are discussed.

## Results

### Effect of ice-binding protein on the survival rate of transgenic *C. elegans* upon cold shock and during freezing

**Figure 1:**
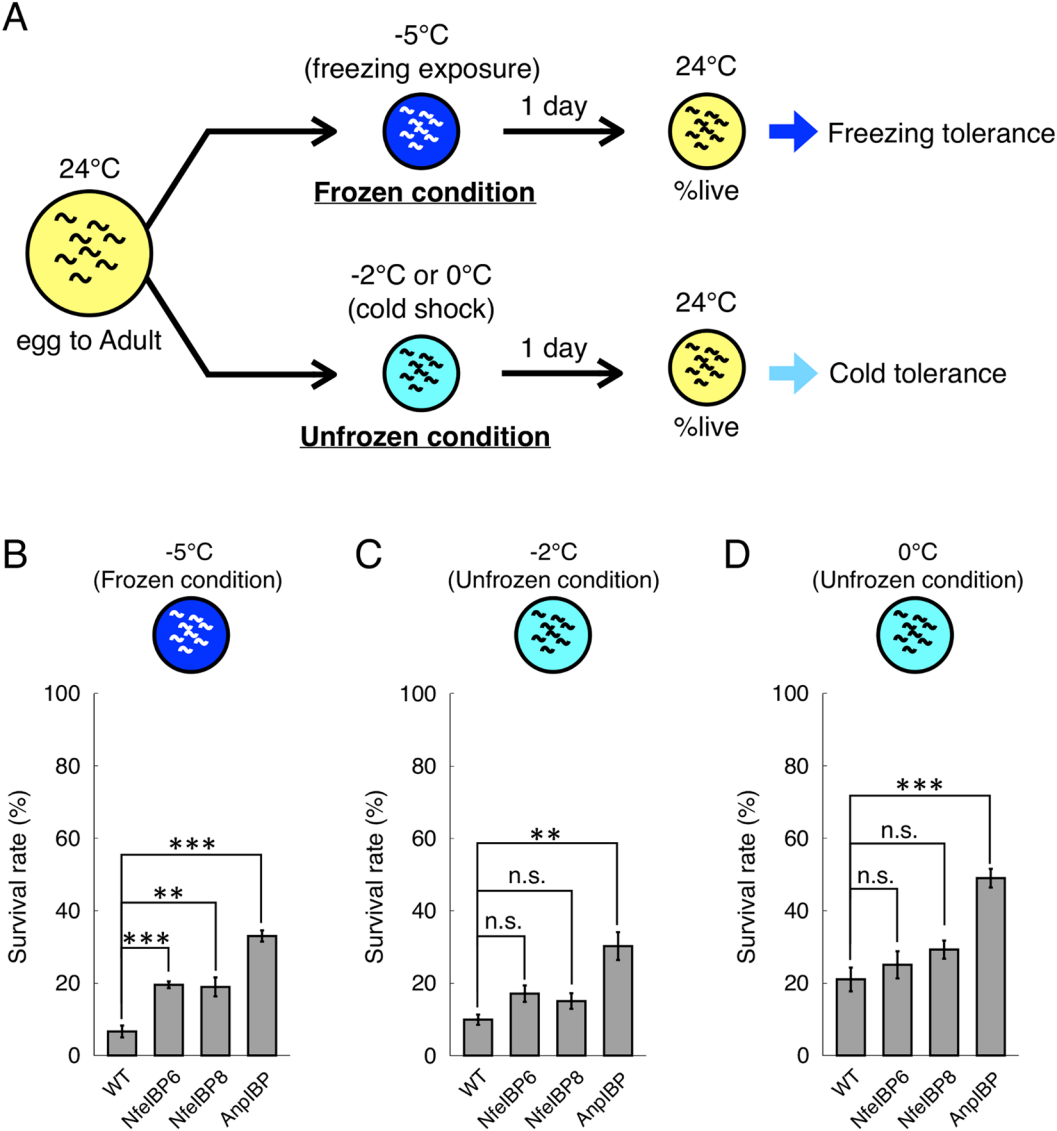
Survival rate of wild-type and IBP-expressing worms after cold shock and freezing. (**A**) Cold- and freezing-tolerance assays to observe the survival rate of *C. elegans*. (**B**,**C**) Survival rate of each IBP-expressing worm after −5 °C (freezing) exposure and −2 °C and 0 °C (cold shock) exposure. NfeIBPs were isolated from *Zoarces elongatus Kner* (Notched-fin eelpout). AnpIBP was isolated from *Antarctomyces psychrotrophicus*. In each assay, n ≧ 20 (group ≧ 5). Error bars indicate the standard error of the mean. A Bonferroni t-test was performed to compare the IBP-expressing worms and wild-type animals. ***p < 0.001, n.s. represents no significance.

IBPs can inhibit ice-crystal growth upon freezing and are thought to stabilize the cellular membrane upon cold shock. These abilities of IBPs are thought to reduce cold- and freezing-induced damage to cells and tissues in living organisms. To elucidate whether several IBPs, i.e., fish NfeIBP6 and NfeIBP8 and fungal AnpIBP1a N55D (AnpIBP), improve cold and freezing tolerance in living animals, we examined the survival rate of transgenic *C. elegans* expressing IBPs in cells after cold shock and freezing (Fig. 1A). The survival rate of several transgenic worms expressing IBPs in neurons or intestinal cells was higher than that of wild-type animals (Fig. S1). However, their survival rate seemed to be lower than that of worms expressing IBPs in body wall muscles (Fig. 1 and S1). The survival rate of the wild-type animals after −5 °C exposure was 6.6%. In contrast, the survival rates of the worms expressing NfeIBP6, NfeIBP8, or AnpIBP in body wall muscles were 19.5%, 18.9%, and 32.9%, respectively (Fig. 1B). The nematode growth medium (NGM) plates used in this assay were frozen at −5 °C (Fig. S2A). The worms also began to freeze upon −5 °C exposure (Fig. S2B). Each IBP likely plays a role in the freezing tolerance of *C. elegans* via ice-growth inhibition. After a −2 or 0 °C cold shock, the survival rates of AnpIBP-expressing worms were also higher than those of the wild-type animals (Fig. 1C,D). During these cold shocks, the NGM plates did not freeze, and the worms did not freeze (Fig. S2A,B). IBPs might work by stabilizing the fluidity of the cell membrane without the IBP-ice interaction. To demonstrate this cellular-protection ability of IBPs, we further examined the survival rate after a 2 or 5 °C cold shock. Although ice crystals are not generated at these hypothermic temperatures, the survival rate of the IBP-expressing worms was significantly higher than that of the wild-type animals (Fig. S3A,B). These results suggest that the ice-growth inhibition of IBPs mainly plays a role in freezing tolerance in *C. elegans* after −5 °C exposure, whereas the cellular-protection functions of IBPs play a role in cold tolerance after a −2 to 5 °C cold shock.

### Mutation in the fungal protein AnpIBP1a reduces its ice-binding ability

**Figure 2:**
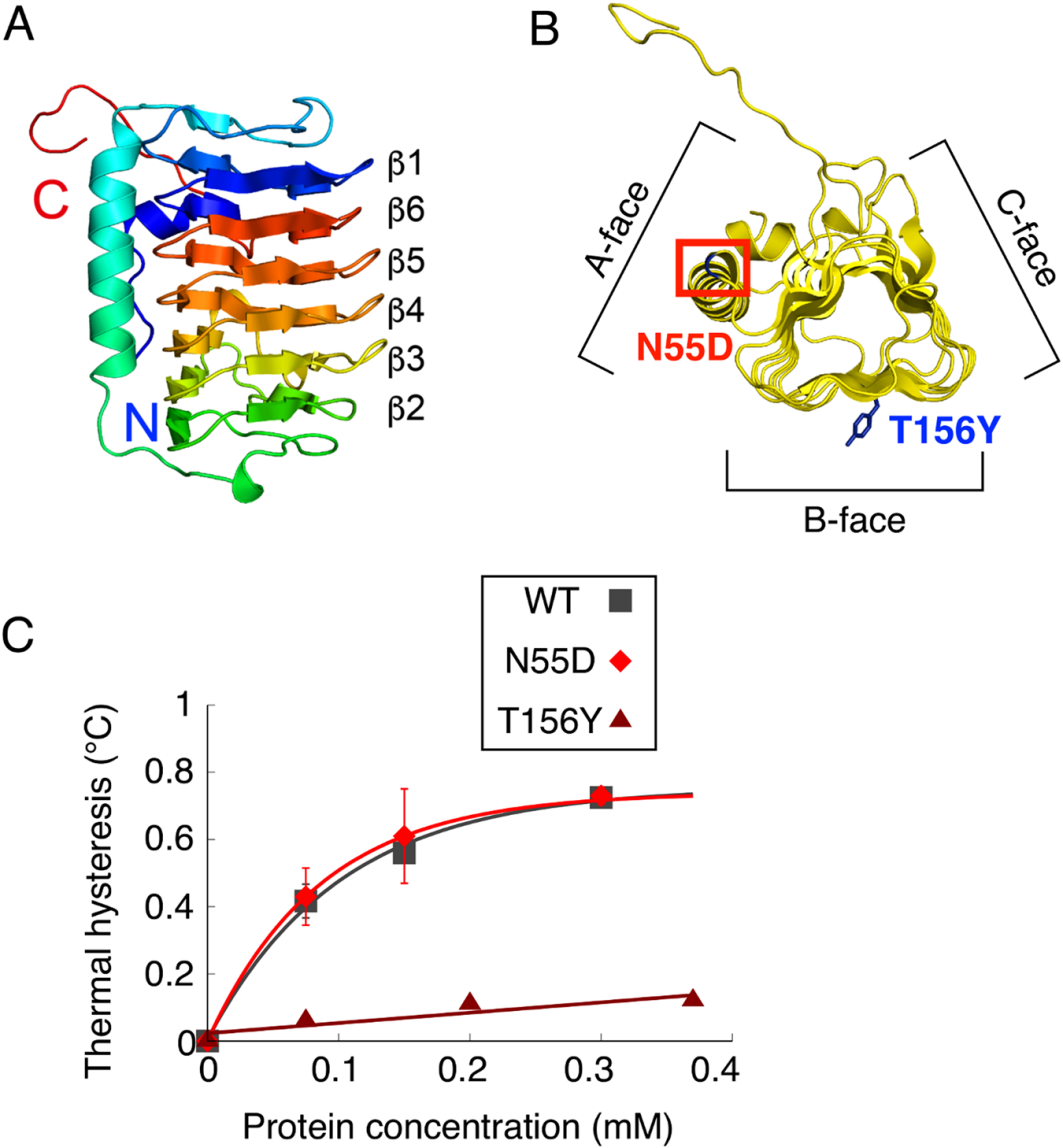
Model structure and TH activities of AnpIBP1a and its mutants. (**A**) Predicted structure of AnpIBP1a coloured from blue (N-terminus) to red (C-terminus). The β-sheets of AnpAFP1a were usually stacked in the order of β1-β6-β5-β4-β3-β2, which is similar to the order in other microbial IBPs, such as TisIBP6^34^. (**B**) Upper view of (**A**). Triangular cross-section showing three parallel β-sheets, namely, A-face, B-face, and C-face. Mutated residues are shown in blue (T156Y) and red (N55D). Tyrosine was used to determine the ice-binding site (IBS), which is known to affect the antifreeze activity. Asn55 was mutated to an aspartic acid residue to remove N-glycan, which plays an unknown role. (**C**) TH activities of AnpIBP1a, AnpIBP1a_N55D, and AnpIBP1a_T156Y plotted as a function of the protein concentration (µM). In each assay, n = 3. Error bars indicate the standard error of the mean. The error bars of AnpIBP1a_T156Y are shorter than the size of the symbol.

**Figure 3:**
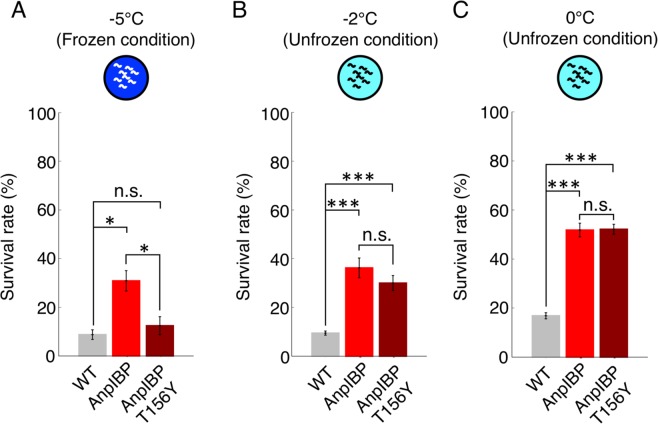
Survival rate of worms expressing AnpIBP and its mutant after cold shock and freezing. (**A**–**C**) Survival rate of each IBP-expressing worm and wild-type worm after −5 °C (freezing) exposure and −2 °C and 0 °C (cold shock) exposure. In each assay, n ≧ 20 (group ≧ 4). Error bars indicate the standard error of the mean. A Bonferroni t-test was performed for multi-comparisons. *p < 0.05, ***p < 0.001, n.s. represents no significance.

The expression of IBPs improved the survival rate of *C. elegans* after each cold shock and freezing. Our results suggest that these tolerances are caused by two abilities of IBPs. One ability is ice-growth inhibition, and other ability is cellular protection. Several studies have reported that these abilities of IBPs are correlated with their TH activity^8,22^. First, to elucidate whether differences in TH activity affect the survival rate of worms after cold shock and freezing, we focused on the fungal protein AnpIBP1a and examined the relationship between its structural properties and TH activity. The overall structure of AnpIBP1a is a right-handed β-helical fold with a parallel α-helix, which is similar to that of other microbial IBPs, such as LeIBP^25^ (Fig. 2A). AnpIBP1a consists of three parallel β-sheets (A-face, B-face and C-face) with a triangular cross section (Fig. 2B). AnpIBP1a possesses an N-glycosylation site at Asn55, which is positioned in the N-terminus of the α-helix and is not present in other microbial IBPs (Fig. 2B). To determine whether this glycan plays an important role in the antifreeze activity of AnpIBP1a, Asn55 was substituted by an aspartic acid residue. The AnpIBP1a N55D mutant exhibited TH activity of 0.7 °C at 300 µM, which is nearly the same as that of AnpIBP1a (Fig. 3C). This result indicates that the N-glycan of AnpIBP1a is not involved in its antifreeze activity. The analysis of the shape correlation following the single residue substitution showed that the β-sheet of the B-face participates in ice binding and that the reduction in the ice-binding ability causes TH loss^26–28^. To examine the ice-binding site of AnpIBP1a, Thr156 on the B-face was mutated to a tyrosine residue (Fig. 2B). The TH activity of the AnpIBP1a WT and N55D mutant proteins was 0.7 °C at 300 µM (Fig. 2C). In contrast, the TH activity of the AnpIBP1a T156Y mutant was decreased to only 0.1 °C at 370 µM. This result suggests that the B-face of AnpIBP1a is an IBS similar to that of other microbial IBPs, such as LpIBP^25^ and TisIBP6^26^.

### TH loss in IBPs causes reduced freezing tolerance in *C. elegans*

The TH activity of AnpIBP is significantly reduced by the mutation (T156Y) on the AnpIBP1a B-face (Fig. 2C). To elucidate whether the difference in TH activity in AnpIBP affects freezing tolerance in *C. elegans*, we compared the survival rates of worms expressing AnpIBP1a N55D (AnpIBP) and AnpIBP1a N55D/T156Y (AnpIBP T156Y). After −5 °C exposure, the survival rate of the worms expressing AnpIBP T156Y was significantly lower than that of those expressing AnpIBP (Fig. 3A). The NGM plates and worms were frozen at −5 °C during the exposure (Fig. S2A,B). AnpIBP likely inhibits ice-crystal growth to promote freezing tolerance in worms. Because the TH activity of the AnpIBP T156Y mutant is only 0.1 °C, the ice-growth inhibition activity of this protein is weak. The survival rate did not significantly differ between the AnpIBP- and AnpIBP T156Y-expressing worms after a 0 or −2 °C cold shock, but the survival rate of the IBP-expressing worms was higher than that of the wild-type animals (Fig. 3B,C). The NGM plates and worms did not freeze at these temperatures (Fig. S2A,B), suggesting that this mutation of AnpIBP does not reduce its cellular protection ability. These results suggest that this T156Y mutation on the IBS of AnpIBP may cause the destruction of cells and tissues in living animals by reducing the IBP-ice interaction.

The ice-binding ability depends on its concentration. The IBP expression levels in each transgenic line are critical for *in vivo* function. To elucidate whether the expression level of the transgenes depends on the survival rate of *C. elegans*, we generated different transgenic lines using the same constructs of NfeIBP6, NfeIBP8, AnpIBP, and AnpIBP T156Y. The copy number and expression level of the transgenes vary among the transgenic lines. We generated multiple lines (#1-#3 or #1-#4) for the 4 IBP constructs and found that the expression level could differ among the different transgenic lines; however, the correlation between TH activity and the survival rate was largely similar regardless of whether the worms were derived from the same strain or different strains expressing the same construct (Fig. S4A). The distributions of the survival rates of the multiple transgenic lines were significantly higher than those of the wild-type (Fig. S4B–D). Interestingly, at −5 °C, the distribution of the survival rate of the NfeIBP8-expressing worms was significantly higher than that of the NfeIBP6-expressing worms (Fig. S4B). The TH activity of the NfeIBP8 protein was higher than that of the NfeIBP6 protein^22^. In addition, the survival rate of the AnpIBP-expressing worms was significantly higher than that of the AnpIBP T156Y-expressing worms (Fig. S4B). The TH activity of the wild-type AnpIBP protein was higher than that of the AnpIBP T156Y mutant (Fig. 2C). However, following the −2 or 0 °C cold shock, the distribution of the survival rate did not significantly differ between the NfeIBP6- and NfeIBP8-expressing worms or between the AnpIBP- and AnpIBP T156Y-expressing worms, respectively (Fig. S4C,D). Interestingly, the survival rate of the AnpIBPs-expressing worms seemed to be higher than that of the fish NfeIBPs-expressing worms (Fig. S4C,D). These results suggest that the expression level of IBPs in different transgenic lines varies but does not dramatically affect the IBP effects. We conclude that ice-binding property-related TH activity is a key factor responsible for freezing tolerance in *C. elegans*.

### Freezing tolerance is improved by hyperactive TisIBP8

**Figure 4:**
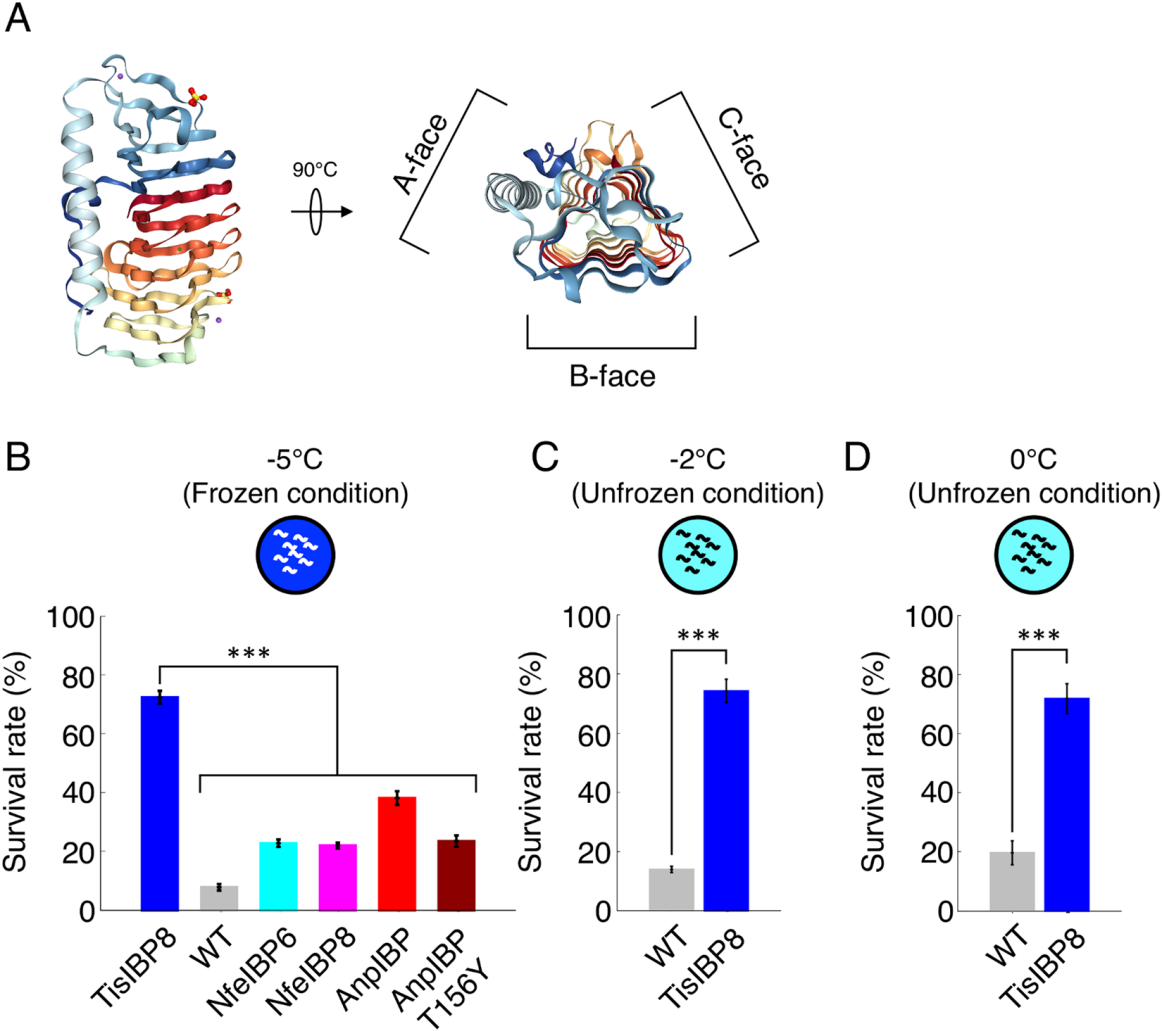
Survival rate of worms expressing TisIBP8 after cold shock and freezing. (**A**) Crystal structure of TisIBP8. The TisIBP8 structure (PDB ID code 5B5H) was visualized using PyMOL^38^. (**B**) Survival rates of TisIBP8-expressing, wild-type, NfeIBP6, NfeIBP8, AnpIBP, and AnpIBP T156Y-expressing worms after a −5 °C exposure. In each assay, n ≧ 20 (group = 5). Error bars indicate the standard error of the mean. A Bonferroni t-test was performed to compare the TisIBP-expressing worms with the other worms. ***p < 0.001. (**C**,**D**) Survival rates of the wild-type and TisAFP8-expressing worms after cold shock (−2 °C and 0 °C). In each assay, n ≧ 20 (group ≧ 5). Error bars indicate the standard error of the mean. Welch’s two-sample t-test (unequal variances t-test) was performed. ***p < 0.001.

A single-residue substitution in AnpIBP resulted in a drastic reduction in the ice-binding ability of this protein (Fig. 2C), resulting in a reduced freezing tolerance in worms (Fig. 3A). TH activity is likely an important parameter involved in the regulation of the tolerance of animals to freezing temperature. Thus, an IBP exhibiting high TH activity may dramatically improve the survival rate of animals after cold shock and freezing. Recently, the basidiomycetes TisIBP was isolated from *Typhula ishikariensis*. TisIBP8 has a high TH activity of 1.9 °C (at 0.12 mM), which is similar to the hyperactive IBP observed in insects^2^, although its crystal structure is similar to that of AnpIBP (Figs 2A and 4A). We further investigated the role of the TH activity of IBPs by observing the survival rate of worms expressing TisIBP8. Surprisingly, after a −5 °C exposure, the survival rate of the TisIBP8-expressing worms was 72.4%, whereas that of the wild-type and transgenic worms expressing NfeIBP6, NfeIBP8, AnpIBP, or AnpIBP T156Y was 7.8%, 22.8%, 22.0%, 38.1%, and 23.5%, respectively (Fig. 4B), suggesting that TisIBP8 dramatically improves the survival rate upon freezing exposure. Furthermore, after a −2 or 0 °C cold shock, the survival rates of the TisIBP8-expressing worms were higher than those of the wild-type animals (Fig. 4C,D). These results suggest that TisIBP8 has high ice-growth inhibition and cellular protection abilities *in vivo*.

### Cellular protection is improved by hyperactive TisIBP8

**Figure 5:**
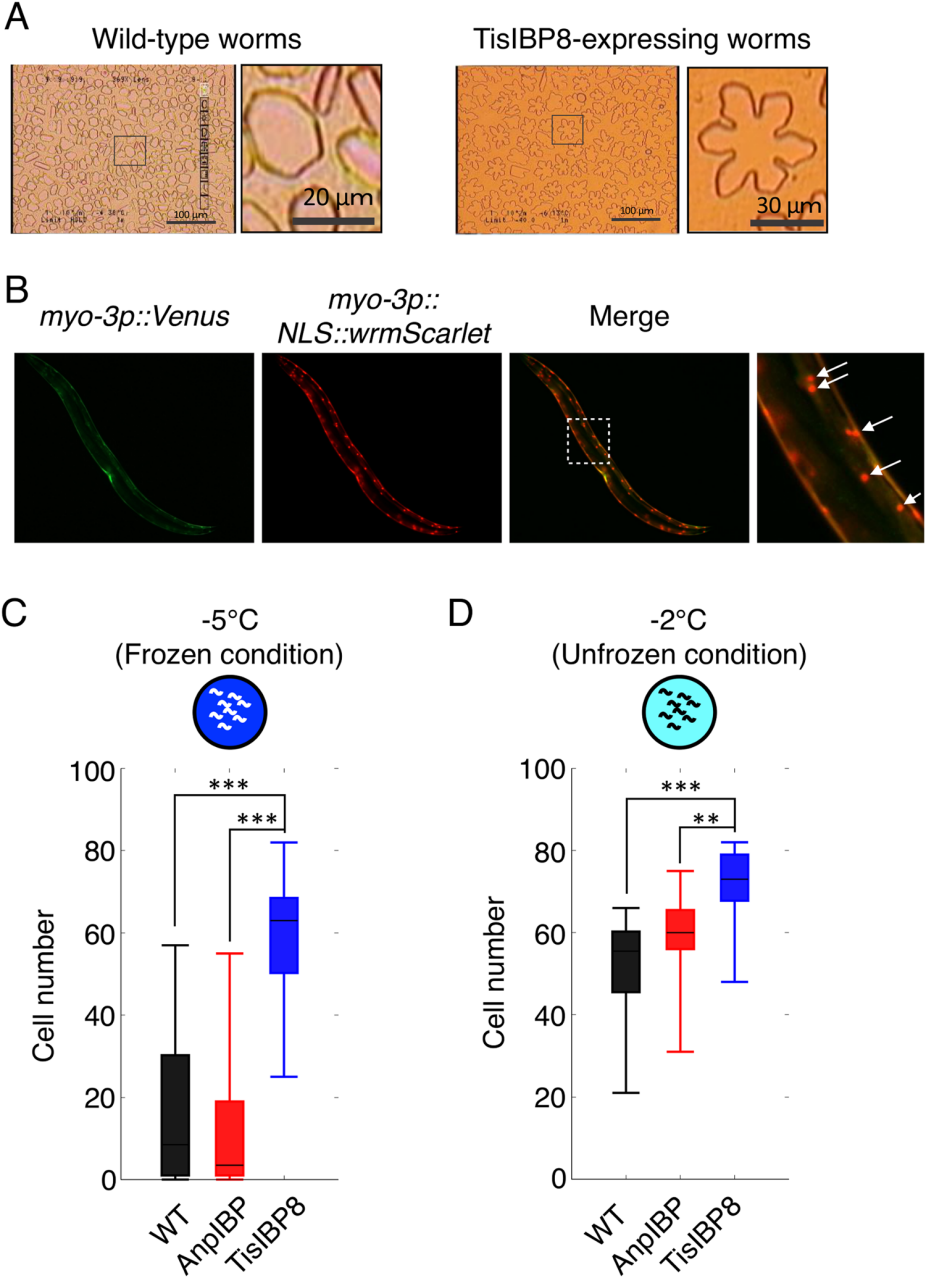
TisIBP8 protects body wall muscle cells in *C. elegans*. (**A**) Ice crystals in extracts from WT and TisIBP8-expressing worms. The ice crystal shape in the WT extracts is hexagonal, whereas that in the TisIBP8-expressing worm extracts is star-like. (**B**) Localization of the wrmScarlet signal in body wall muscles. The panel on the right shows an enlarged view of the dotted square in the merged panel. The arrows indicate wrmScarlet puncta from the cell nucleus in body wall muscles. (**C**,**D**) Number of cells expressing wrmScarlet in body wall muscle cells in wild-type, AnpIBP and TisIBP8 worms. n ≧ 20. Boxes show the median and first and third quartiles. The Wilcoxon rank sum test was performed. **p < 0.01, ***p < 0.001.

Worms expressing TisIBP8 in the body wall muscles showed increased cold and freezing tolerance to 0 °C and −2 °C cold shock and to −5 °C freezing. These results suggest that intracellular TisIBP8 in body wall muscles contributes to improving the survival rate of TisIBP8-expressing *C. elegans*. We first confirmed whether TisIBP8 had ice-growth inhibition activity within transgenic *C. elegans*. The TisIBP8 protein extracts obtained from the transgenic worms were capable of modifying ice crystals into a star-like shape with a six-fold symmetry^29,30^, whereas the ice crystals in the extracts from the wild-type animals exhibited a hexagonal shape (Fig. 5A). These results indicate that TisIBP8 proteins in worms have the ability to modify and inhibit the formation of ice crystals.

TisIBP8 in *C. elegans*, which modifies the ice-crystal shape (Fig. 5A), might protect the cells in living worms from exposure to freezing temperatures. In addition, several IBPs can protect the extracellular surface of cells under hypothermic conditions^7,8,31^. The cellular protection mechanism of IBPs might be the stabilization of the structure of cell membrane lipids upon cold shock. After exposure to cold temperatures, cells are destroyed through leaks (the cellular contents leak out)^6,32–34^ that likely result from the inability of these cells to maintain their shape. Here, we examined whether TisIBP8 protects *C. elegans* cells from the intracellular side during cold shock and freezing. Using transgenic worms expressing the fluorescent protein wrmScarlet^35^ in the muscles in a nucleus-bound fashion, we evaluated the ability of IBPs to protect cells by comparing the number of cells in the wild-type worms and worms expressing AnpIBP and TisIBP8. The wrmScarlet is localized at the cell nucleus. The individual muscle cells are easily identified as small fluorescent puncta (Fig. 5B and S5A). If the muscle cells could not keep their shape upon cold shock and freezing, the cell nucleus should also be broken. In fact, the cell number (wrmScarlet puncta) in the wild-type animals appeared to decrease in response to a decrease in temperature (Fig. S5B,C). The median cell number in the TisIBP8-expressing worms was approximately 59, whereas that in the wild-type and AnpIBP-expressing worms was approximately 17 and 12, respectively, after a −5 °C freezing exposure (Fig. 5C). Similar results were observed in worms exposed to a −2 °C cold shock (Fig. 5D). In addition, AnpIBP protected the muscle cells (Fig. S5C), although its ability to protect cells was lower than that of TisIBP8. These results suggest that TisIBP8 protects muscle cells, resulting in improved freezing and cold tolerance in worms.

## Discussion

In the current study, we investigated the effects of IBPs on cold and freezing tolerance in living *C. elegans*. We found that IBPs can improve the survival rate of worms and protect their cells from cold and freezing temperatures. IBPs can inhibit ice-crystal growth to achieve freezing tolerance. The effect *in vivo* is associated with the TH activity of the IBPs. The specific binding of IBPs to ice is closely related to the TH activity. AnpIBP only binds the prism planes of ice crystals and not the basal planes^2,23^, whereas TisIBP binds both the prism and basal planes of ice crystals^26^. Ice crystals rapidly grow into needle-like shapes once the freezing hysteresis exceeds the TH activity. Needle-like ice crystals are generated by AnpIBP binding only the prism planes of the ice crystals and may penetrate and destroy the cell membrane at freezing temperatures^34^. The AnpIBP-expressing worms likely experienced cell membrane damage due to needle-like ice. How different IBPs bind ice may affect their ability to protect cells and confer freezing tolerance to *C. elegans*.

The cell membrane (lipid bilayer) changes from liquid to gel at cold temperatures, resulting in the leakage of the intracellular contents, subsequently leading to cell destruction^6,32–34^. IBPs can inhibit the leakage of cellular contents across the cell membrane^36,37^. In addition to inhibiting ice growth, IBPs likely bind the cell membrane in *C. elegans*, preventing cell leakage (cellular contents leaking out) caused by the phase transition of the cell membrane. This membrane stabilization effect of IBPs likely improved the survival rate of *C. elegans* during the −2 to 5 °C cold shock. To achieve this membrane protection activity, IBPs should be able to bind the cell membrane. The results of several studies investigating IBP-ice interactions suggest that a polypentagonal ice-like water network at ice-binding sites binds a quasi-liquid layer of ice crystal, resulting in the formation of an IBP-ice crystal complex. This ordered water molecular network, which rapidly and completely regulates the ice-binding process, can fit the quasi-liquid layer and may exist on ice-binding residues with favourable arrangements in IBPs. One possible explanation for the cellular protection conferred by IBPs is that water molecules arranged on the *C. elegans* cell membrane form an ordered hexagonal structure as a quasi-liquid layer allowing IBPs exhibiting high TH activity to strongly bind worm cell membranes through an ordered hexagonal water arrangement. The increased stability of cell membranes and TH activity, including the specific mode of ice binding, may be key factors responsible for cold- and freezing-tolerance under cold conditions. In this study, we did not examine the details of IBP-membrane interaction. Our future studies should provide a better understanding of the relationship between the cell membrane protection function and IBP characteristics, including IBP expression levels and its interaction with cell membranes, using several transgenic worm strains, such as those expressing intra- or extra-cellular IBP. We believe that these *in vivo* analyses could shed light on the mechanisms through which ice crystals interact with cell membranes, resulting in leaky contents and the ability to maintain a supercooled state dependent on TH activity in living animals.

The proposed genetic expression approach could be of interest for high-performance preservation as IBP cold preservation could be applied to many materials ranging from various industrial materials, such as raw foods, to medical materials, such as cells and tissues. Our investigation using molecular genetic manipulation of living *C. elegans* worms may be a step towards establishing a simple preservation method involving the freezing of fresh living cells.

## Methods

### Structural modelling of AnpIBP1a

The 3D structure of AnpIBP1a was developed using the software MODELLER (http://salilab.org/modeller/) with the following structures in the PDB as templates: TisIBP8 (PDB ID = 5B5H), LeIBP (3UYU), FfIBP (4NU2), ColIBP (3WP9), and IBPv_a (5UYT). The AnpIBP1a structure was visualized using PyMOL^38^.

### Expression and purification of recombinant AnpIBP1a and its mutants

cDNA encoding AnpIBP1a (accession number LC378707 in DDBJ/EMBL/GenBank) was inserted into the plasmid pPICZα from an EasySelectTM Pichia Expression Kit (Invitrogen Co., Calif., U.S.A.) using the restriction enzymes *Xho*I and *Not*I. Before the transformation, an expression vector containing the AnpIBP1a gene was linearized with *Pme*I. The transformation of the *Pichia pastoris* X33 strain was performed using a Pichia EasyComp Transformation Kit (Thermo Fisher Scientific, MA, USA). One resulting transformant was precultured in 80 mL of BMGY medium (buffered glycerol-complex medium; 1% yeast extract, 2% peptone, 100 mM potassium phosphate (pH 6.0), 1.34% YNB, 4 × 10^−5^% biotin and 1% glycerol) for 3 days at 22 °C. Once the OD600 of the culture reached 10, the cells were harvested by centrifugation at 7,500 rpm for 10 min at 20 °C and then resuspended in 450 mL of 2 × BMMY medium (buffered methanol-complex medium; 1% yeast extract, 2% peptone, 100 mM potassium phosphate (pH 6.0), 1.34% YNB, 4 × 10^−5^% biotin and 0.5% methanol). The cells were cultivated in a 1-L BMJ-01P fermenter (ABLE, Tokyo, Japan) for 5 days at 20 °C. Methanol was continuously added to the fermenter through a peristaltic pump at a flow rate of 0.5–3.5 mL/h. After 5 days of cultivation, the cells were removed by centrifugation at 7,500 rpm for 10 min at 4 °C, and the culture supernatant was dialyzed against buffer A (20 mM Tris-HCl buffer (pH 8.0) and 0.5 M NaCl). The dialysate was loaded onto a 30 mL Ni-NTA Superflow column (QIAGEN GmbH, Germany) equilibrated with buffer A, and then, the bound protein was eluted with buffer A containing 200 mM imidazole. The eluted fraction was dialyzed against 20 mM Tris-HCl buffer (pH 8.0). Subsequently, the dialyzed sample was applied to a High Q column (Bio-Rad, CA, U.S.A.), and the bound protein was eluted with a 0–300 mM NaCl gradient. The antifreeze-active fractions were recovered and loaded onto a Superdex-200 gel-filtration column (GE-Healthcare, Amersham, UK) equilibrated with buffer A. The antifreeze-active fractions were recovered, dialyzed against water, and frozen at −20 °C until use.

### TH measurements

The TH activities of AnpIBP1a and its mutants were measured using a photomicroscope system following an established protocol^39^. IBP was dissolved in 20 mM Tris-HCl buffer (pH 8.0). The sample (1 µL) was rapidly cooled in a glass capillary until frozen and then slowly melted until a single ice crystal formed. The melting point (Tm) is reported as the temperature at which the ice crystal began to melt. The single ice crystal was maintained at Tm − 0.1 °C for 5 min and then cooled again at a rate of 0.1 °C/min. The non-equilibrium freezing point (Tf) of the solution is reported as the temperature at which the ice crystal grew rapidly. The TH values at each concentration of the IBPs are reported as the difference between Tm and Tf (i.e., TH = |Tf − Tm|).

### Molecular biology

We used the *myo-3*, rimb-1 (*H20)*, and *elt-2* promoter regions for the specific expression of IBPs in body wall muscles, neurons, and intestinal cells, respectively. The region in each promoter was PCR amplified and inserted at the *Xba*I and *Apa*I sites of the plasmid pPD95.79 (a kind gift from Andrew Fire). The coding sequences of the IBPs were PCR amplified using the following primer sets:

*NfeIBP6*: 5′aaagGAATTCATGGGCGAGTCCGTGGTGGC3′ and 5′atgcGAATTCCTACTTTGCCGGGACGTACG3′; *NfeIBP8*: 5′atgcGAATTCATGAACCAGGCGTCCGTGGT3′ and 5′aagcGAATTCCTAAGCCGGGGTGTACCCTT3′; *AnpIBP1a N55D*: 5′ggggGAATTCATGACTGGTTTGGATTTGGGTGC3′ and 5′gggcGAATTCCTAAACCTTGAAGAACTTAG3′; and *TisIBP8*: 5′cggcGAATTCGAAGGAGATATACATATGGCTGGT3′ and 5′ataaGAATTCCTCGAGTGCGGCCGCCTATTTTTGC3′. An AnpIBP1a N55D/T156Y fragment was created by overlap PCR fusion using the AnpIBP N55D primers listed above and the following primers: 5′atgcAGTTCCCAAATAAGCGGAGG3′ and 5′atgcCCTCCGCTACTTTGGGAACT3′. Each IBP fragment was inserted between the *EcoRI* sites of the *myo-3p::Venus* construct. For the cell counting analyses, the *NLS::wrmScarlet* construct was PCR amplified using the following primers: 5′agttGGTACCatgccaaagaagaagcgtaaggtcA3′ and 3′atgaGAATTCgctcgccaaggaggtcgagaactag5′.

The *NLS::wrmScarlet* PCR product was replaced with *Venus* using the *Kpn*I and *Eco*RI sites of the *myo-3p::Venus* construct.

### Germline transformation

To generate the transgenic animals, the resulting plasmids were injected into *lin-15* (*n765ts*) mutant animals at a concentration of 5–100 ng/uL with a pbLH98 *lin-15* (+) injection marker using a standard microinjection method^40^. The marker DNA rescues the multivulva phenotype in the *lin-15* mutant animals. The plasmid DNA rearranges to form extrachromosomal arrays that are stably inherited although not at the same efficiency as actual chromosomes^41^. Extrachromosomal arrays have been estimated to contain hundreds of copies of injected plasmids^40,42^. The details of the construction of the transgenic worms are shown in Table S1.

### *C. elegans* cold- and freezing-tolerance assay

Well-fed animals were used for the cold- and freezing-tolerance assay. The worms were cultivated from eggs to the adult stage at 24 °C on nematode growth medium (NGM) plates with sufficient food *Escherichia coli* OP50. Twenty to fifty adult worms were placed on the test plates. These adult worms were cultivated for 1 day under each cold shock (−2, 0, 2 and 5 °C) or freezing conditions (−5 °C), and the plates were subsequently transferred to room temperature (RT). After 2 hours at RT, the numbers of dead and living animals in the plate were counted, based on movement in response to touch stimulus^43^. A Bonferroni t-test was performed for multi-comparison. Student’s t-test was performed to compare two samples. If one of the two samples had unequal variances, Weltch’s t-test was performed. All statistical analyses were performed using R version 3.3.1.

### Cell counts in the body wall muscles of *C. elegans*

Cells and tissues are easily visualized under a microscope because *C. elegans* has a transparent body. To demonstrate the effects of IBPs at the cellular resolution level in living worms, we used transgenic worms expressing the fluorescent protein wrmScarlet in muscle cells in a nucleus-bound fashion. The wrmScarlet localizes to the cell nucleus via nuclear localization signal (*NLS*) as small puncta (Fig. 5B). The expression of wrmScarlet facilitates the identification of individual cells in living *C. elegans* (Fig. 5B and S5A). Following cold and freezing exposure, wrmScarlet puncta were counted under the same conditions used in the tolerance assay. Adult worms were mounted on a 2.0% agarose pad with 50 mM sodium azide in M9 solution for anaesthesia. The images were acquired under a fluorescence microscope (Zeiss, AxioImager. A2) with a 40× objective lens.

The number of cells counted in the WT and AnpIBP worms did not satisfy a Gaussian distribution based on the Shapiro-Wilk test. Therefore, a non-parametric Wilcoxon rank sum test was performed to evaluate the median difference in the cell number using R version 3.3.1.

### Time-lapse observation of freezing in *C. elegans* while cooling on a cold stage

One- or two-day-old adult worms were used for this observation following an established protocol^13^. The worms were mounted on a 2.0% agarose pad with DDW. The agarose pad was set on the stage of a bright-field microscope (Olympus BX50) with a 10× objective lens. A heating/cooling stage (Model number: 10021, Japan High Tech) mounted on the microscope was used to hold the agarose pad at −2 or −5 °C. Time-lapse images were obtained using an ORCA-Flash 4.0 CCD camera (Hamamatsu Photonics) controlled by HCImage software (Hamamatsu Photonics). The images were captured at a rate of 5 frames/sec.

## Supplementary information

Supplementary Information

## Data Availability

Strains are available upon request.
