# Supplementary material for: Expression of Ice-Binding Proteins in Caenorhabditis elegans Improves the Survival Rate upon Cold Shock and during Freezing
Source: Sci Rep. 2019 May 15;9:6246. doi: 10.1038/s41598-019-42650-8 (PMC6520345; doi:10.1038/s41598-019-42650-8)
Supplement: Supplementary file 1 — Supplementary Information [file 41598_2019_42650_MOESM1_ESM.docx]

Supplementary Information for

Expression of Ice-Binding Proteins in Caenorhabditis elegans Improves the Survival Rate upon Cold Shock and during Freezing

Masahiro Kuramochi, Chiaki Takanashi, Akari Yamauchi, Motomichi Doi, Kazuhiro Mio, Sakae Tsuda, and Yuji C. Sasaki

Masahiro Kuramochi

Email: [masahiro-kuramochi@edu.k.u-tokyo.ac.jp](mailto:masahiro-kuramochi@edu.k.u-tokyo.ac.jp)

Sakae Tsuda

Email: [s.tsuda@aist.go.jp](mailto:s.tsuda@aist.go.jp)

Yuji C. Sasaki

Email: [ycsasaki@edu.k.u-tokyo.ac.jp](mailto:ycsasaki@edu.k.u-tokyo.ac.jp)

**This file includes:**

Figs. S1 to S5

Tables S1

**Figure S1. Survival rate of wild-type and transgenic worms expressing IBP in neurons or intestinal cells specifically after cold shock and freezing.** (A-E) Survival rate of each IBP-expressing worm and wild-type worm after -5°C (freezing) exposure and -2°C, 0°C, 2°C and 5°C (cold shock) exposure. In each assay, n ≧ 20 (group ≧ 4). Error bars indicate the standard error of the mean. A Bonferroni t-test was performed to compare the IBP-expressing worms and wild-type animals. **p < 0.01, ***p < 0. 001.

**
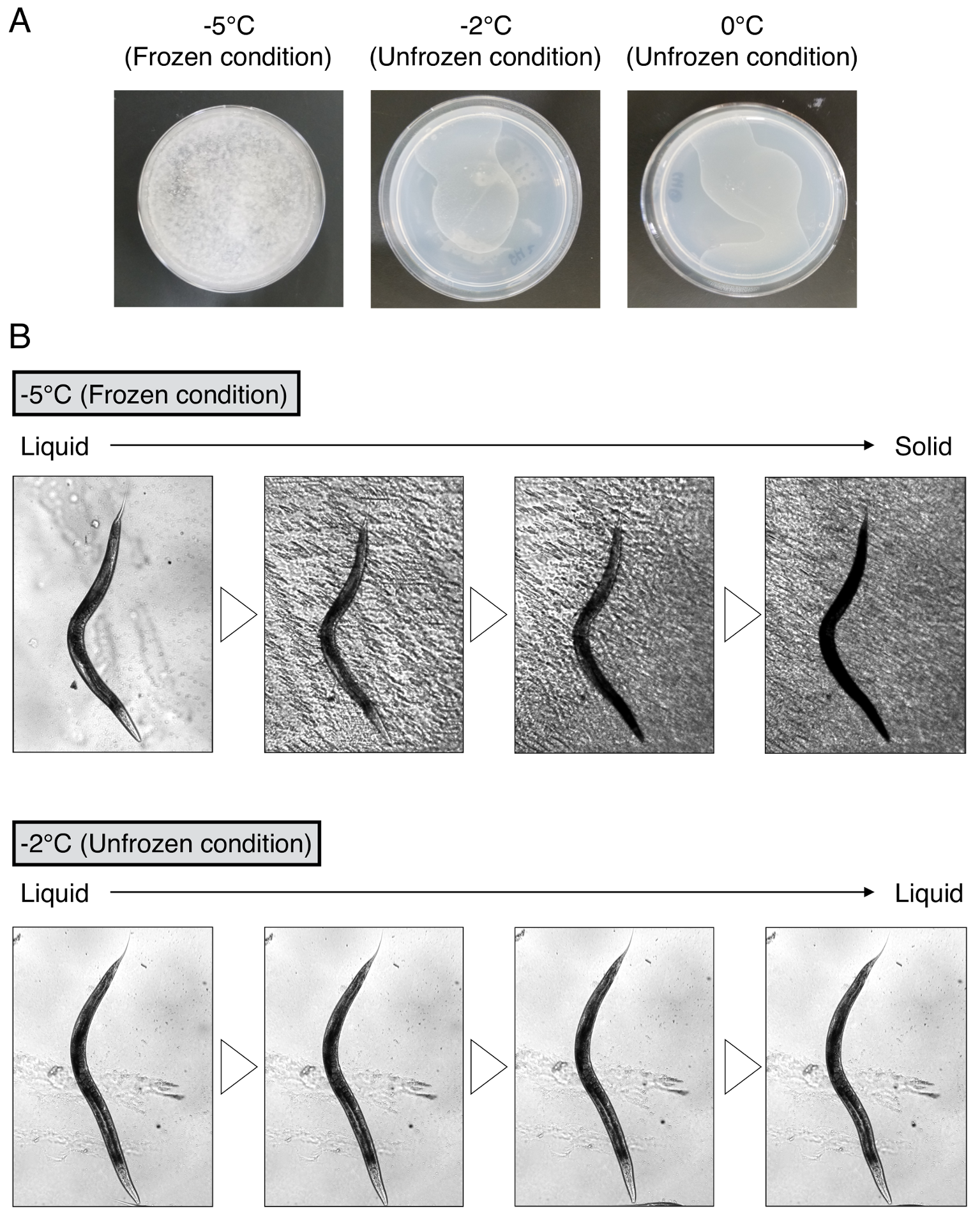
**

**Figure S2. Observation of freezing under our assay conditions in *C. elegans*.** (A) NGM plates used for the cold tolerance assay were frozen upon the -5°C exposure, whereas the plates did not freeze upon the -2 or 0°C exposure. (B) Time-lapse observation of the freezing process in *C. elegans* while cooling on a cold stage. Worms exposed to -5°C froze after several minutes, whereas those exposed to -2°C or higher temperatures did not freeze.

**Figure S3. Survival rate of wild-type and IBP-expressing worms after cold shock.** (A, B) Survival rate of each IBP-expressing worm after 2 and 5°C cold shock. In each assay, n ≧ 20 (group ≧ 5). Error bars indicate the standard error of the mean. Bonferroni t-test was performed to compare the IBP-expressing worms and wild-type animals. *p < 0.05, **p < 0.01, ***p < 0.001.

**Figure S4. Survival rate of wild-type (WT) and IBP-expressing worms using multiple transgenic lines after cold shock and freezing.** (A) Survival rate of WT and each IBP-expressing worm after -5°C (freezing) exposure and -2°C and 0°C (cold shock) exposure. In each assay, n ≧ 15 (group ≧ 3). Error bars indicate the standard error of the mean. The error bar of WT is shorter than the size of the symbol. (B-D) Distribution of the survival rate of the WT and IBP-expressing worms in (A). Boxes represent the median and first and third quartiles. These data did not show gaussian distribution based on the Shapiro-Wilk test. Thus, a non-parametric Steel-Dwass test was performed for multi-comparisons. *p < 0.05, **p < 0.01, ***p < 0.001.

**Figure S5. Fungal AnpIBP can protect cells in body wall muscles in *C. elegans*.** (A) Transgenic worms expressing fluorescent protein without NLS-tag (left) and with NLS-tag (right). Small puncta in the figure (right) indicate the cell nuclei in body wall muscles. These wrmScarlet signals were counted after cold and freezing exposure. (B) Localization of the wrmScarlet signal in body wall muscle cells in wild-type and AnpIBP-expressing worms after each cold shock or freezing. (C) The number of cells exhibiting wrmScarlet signals in body wall muscles. In each assay, n ≧ 20. Error bars indicate the standard error of the mean. Student’s t-test was performed to compare the AnpIBP-expressing worms and wild-type animals. *p < 0.05.

**Table S1. Strain list.**

| Strain name | Genotype | Construct (injection concentration) | Fig. |
| --- | --- | --- | --- |
| CMS2 | *kmcEx2;*  *lin-15(n765ts)X* | *myo-3p::Venus::NfeIBP6* (50 ng/uL)  *pbLH98* (*lin-15*(*+*)) (40 ng/uL) | 1B-D  4B  S3  S4 |
| CMS3 | *kmcEx3;*  *lin-15(n765ts)X* | *myo-3p::Venus::NfeIBP8* (50 ng/uL)  *pbLH98* (*lin-15*(*+*)) (40 ng/uL) | 1B-D  4B  S3  S4 |
| CMS4 | *kmcEx4;*  *lin-15(n765ts)X* | *myo-3p::Venus::AnpIBP* (40 ng/uL)  *pbLH98* (*lin-15*(*+*)) (40 ng/uL) | 1B-D  3A-C  4B  S3  S4 |
| CMS5 | *kmcEx5;*  *lin-15(n765ts)X* | *myo-3p::Venus::TisIBP* (30ng/uL)  *pbLH98* (*lin-15*(*+*)) (30ng/uL) | 4B-D  S2B |
| CMS15 | *kmcEx15;*  *lin-15(n765ts)X* | *myo-3p::Venus::AnpIBP T156Y* (30ng/uL)  *pbLH98* (*lin-15*(*+*)) (30ng/uL) | 3A-C  4B  S4 |
| CMS14 | *kmcEx14;*  *lin-15(n765ts)X* | *myo-3p::NLS::wrmScarlet* (20 ng/uL)  *pbLH98* (*lin-15*(*+*)) (30 ng/uL) | 5A, 5D  S5 |
| CMS13 | *kmcEx13;*  *lin-15(n765ts)X* | *myo-3p::Venus::AnpIBP* (30 ng/uL)  *myo-3p::NLS::wrmScarlet* (20 ng/uL)  *pbLH98* (*lin-15*(*+*)) (30 ng/uL) | 5C, 5D  S5 |
| CMS26 | *kmcEx26;*  *lin-15(n765ts)X* | *myo-3p::Venus::TisIBP* (30ng/uL)  *myo-3p::NLS::wrmScarlet* (20 ng/uL)  *pbLH98* (*lin-15*(*+*)) (30ng/uL) | 5A-D |
| CMS6 | *kmcEx6;*  *lin-15(n765ts)X* | *H20p::Venus::NfeIBP6* (40ng/uL)  *pbLH98* (*lin-15*(*+*)) (40ng/uL) | S1 |
| CMS7 | *kmcEx7;*  *lin-15(n765ts)X* | *H20p::Venus::NfeIBP8* (50ng/uL)  *pbLH98* (*lin-15*(*+*)) (40ng/uL) | S1 |
| CMS8 | *kmcEx8;*  *lin-15(n765ts)X* | *H20p::Venus::AnpIBP* (60ng/uL)  *pbLH98* (*lin-15*(*+*)) (40ng/uL) | S1 |
| CMS9 | *kmcEx9;*  *lin-15(n765ts)X* | *elt-2p::WrmScarlet::NfeIBP6* (50ng/uL)  *pbLH98* (*lin-15*(*+*)) (30ng/uL) | S1 |
| CMS11 | *kmcEx11;*  *lin-15(n765ts)X* | *elt-2p::WrmScarlet::AnpIBP* (65ng/uL)  *pbLH98* (*lin-15*(*+*)) (30ng/uL) | S1 |
| CMS38 | *kmcEx38;*  *lin-15(n765ts)X* | *myo-3p::Venus::NfeIBP6* (100ng/uL)  *pbLH98* (*lin-15*(*+*)) (30ng/uL) | S4 |
| CMS39 | *kmcEx39;*  *lin-15(n765ts)X* | *myo-3p::Venus::NfeIBP6* (5ng/uL)  *pbLH98* (*lin-15*(*+*)) (30ng/uL) | S4 |
| CMS40 | *kmcEx40;*  *lin-15(n765ts)X* | *myo-3p::Venus::NfeIBP8* (5ng/uL)  *pbLH98* (*lin-15*(*+*)) (30ng/uL) | S4 |
| CMS41 | *kmcEx41;*  *lin-15(n765ts)X* | *myo-3p::Venus::NfeIBP8* (100ng/uL)  *pbLH98* (*lin-15*(*+*)) (30ng/uL) | S4 |
| CMS42 | *kmcEx42;*  *lin-15(n765ts)X* | *myo-3p::Venus::AnpIBP* (100ng/uL)  *pbLH98* (*lin-15*(*+*)) (30ng/uL) | S4 |
| CMS12 | *kmcEx12;*  *lin-15(n765ts)X* | *myo-3p::Venus::AnpIBP* (5ng/uL)  *pbLH98* (*lin-15*(*+*)) (30ng/uL) | S4 |
| CMS43 | *kmcEx43;*  *lin-15(n765ts)X* | *myo-3p::Venus::AnpIBP T156Y* (5ng/uL)  *pbLH98* (*lin-15*(*+*)) (30ng/uL) | S4 |
| CMS44 | *kmcEx44;*  *lin-15(n765ts)X* | *myo-3p::Venus::AnpIBP T156Y* (100ng/uL)  *pbLH98* (*lin-15*(*+*)) (30ng/uL) | S4 |
